# The role of benign joint hypermobility in the pain experience in Juvenile Fibromyalgia: an observational study

**DOI:** 10.1186/1546-0096-10-16

**Published:** 2012-06-15

**Authors:** Tracy V Ting, Philip J Hashkes, Kenneth Schikler, Anjali M Desai, Steven Spalding, Susmita Kashikar-Zuck

**Affiliations:** 1 Division of Rheumatology, MLC 4010, Cincinnati Children's Hospital Medical Center, 3333 Burnet Avenue, Cincinnati, OH, 45229, USA; 2 Pediatric Rheumatology Unit, Shaare Zedek Medical Center, Jerusalem, Israel; 3 Division of Pediatric Rheumatology, University of Louisville, Louisville, KY, USA; 4 Division of Behavioral Medicine and Clinical Psychology, Cincinnati Children's Hospital Medical Center, Cincinnati, OH, USA; 5 Center of Pediatric Rheumatology, Cleveland Clinic, Cincinnati, OH, USA

**Keywords:** Juvenile fibromyalgia, Hypermobility, Tender point, Pediatric, Pain

## Abstract

**Background:**

Juvenile Fibromyalgia (JFM) is characterized by chronic widespread musculoskeletal pain and approximately 40% of children and adolescents with JFM also suffer from benign joint hypermobility (HM). It is not currently known if the presence of HM affects the pain experience of adolescents with JFM. The objective of this study was to examine whether there were any differences in self-reported pain intensity and physiologic pain sensitivity between JFM patients with and without joint HM.

**Methods:**

One hundred thirty-one adolescent patients with JFM recruited from four pediatric rheumatology clinics completed a daily visual analogue scale (VAS) pain rating for one week and underwent a standardized 18-count tender point (TP) dolorimeter assessment. Medical records were reviewed for the presence of joint HM. Average pain VAS ratings, tender point count and tender point sensitivity were compared between JFM patients with and without hypermobility (HM+ and HM-).

**Results:**

Nearly half (48%) the sample of JFM patients were found to be HM+. HM+ and HM- patients did not differ in their self-reported pain intensity. However, HM + patients had significantly greater pain sensitivity, with lower TP thresholds (p = 0.002) and a greater number of painful TPs (p = 0.003) compared to HM- patients.

**Conclusion:**

The presence of HM among adolescent patients with JFM appears to be associated with enhanced physiologic pain sensitivity, but not self-report of clinical pain. Further examination of the mechanisms for increased pain sensitivity associated with HM, especially in adolescents with widespread pain conditions such as JFM is warranted.

## Background

Juvenile fibromyalgia (JFM) is a chronic condition of widespread musculoskeletal pain and fatigue in children and adolescents. Prevalence estimates for JFM range from 1-6% of the pediatric population [1,2]. While there are often associated symptoms of poor sleep, fatigue, and emotional distress in JFM, pain is the defining component in the diagnosis of fibromyalgia (FM). The report of diffuse pain for 3 or more months and severe pain in multiple tender points upon palpation is required as part of the classification criteria for FM by both Yunus and Masi [3] and the 1990 American College of Rheumatology (ACR) [4] criteria.

Benign joint hypermobility (HM) is a relatively more common condition than JFM, with prevalence rates in children and adolescents estimated to be up to 30% [5]. Children and adolescents with increased joint laxity have been found to frequently suffer from chronic musculoskeletal pain complaints [5,6], although one large population study indicated no such association [7]. Yet many children with joint HM do not suffer from the full spectrum of JFM symptoms. On the other hand, there appears to be a much closer overlap among patients with JFM and benign joint HM. In fact, two studies have reported a higher prevalence of HM co-occurring with JFM. One study found that 81% of Israeli JFM school-children had HM [8], and another study based in the United States reported that 40% of JFM adolescents also had HM [9]. In the adult literature, it has been suggested that the presence of HM is associated with increased pain in women with FM [10]. However, the relationship between pain characteristics and HM has not been examined in children and adolescents with JFM.

The underlying mechanisms for pain hypersensitivity in FM have been extensively studied while the etiology of pain in HM has received little attention. It has been well documented that persons with FM have an overall lower threshold for pain as demonstrated by increased responsiveness and hypersensitivity to pain [11] in the form of central sensitization and wind-up in response to repeated noxious stimulation [12]. It is not currently known whether joint laxity/HM is associated with enhanced sensitization to pain in FM although it has been suggested (though not proven) that repeated microtrauma occurring among persons with abnormal joint hyperextensibility might lead to persistent localized pain [13].

As part of the screening for a larger clinical trial examining the effectiveness of cognitive-behavioral therapy for JFM, we assessed baseline pain intensity and tender point sensitivity (tender point count and tender point threshold) of over 100 adolescents with JFM [14]. For the current study, we reviewed patients’ medical charts to determine the frequency of benign joint HM as determined by their treating rheumatologist. The primary objectives of this study were to examine the prevalence of HM in this clinical sample of adolescents with JFM and to compare the pain experience between JFM patients with joint HM (HM+) and without (HM-). Based upon previous studies, it was anticipated that at least 40% of JFM patients would be HM + [2,5,6,15]. We also hypothesized that JFM patients who were HM +would report higher clinical pain intensity (based upon self-report) and demonstrate enhanced pain sensitivity (based upon dolorimetry) compared to those who were HM-.

## Methods

### Participants

Participants were 131 adolescents (92.4% female, 89.3% Caucasian) with JFM between the ages of 11 and 18 years (mean age = 15.1 years) who were initially screened for the parent clinical trial. Participants were recruited from four pediatric rheumatology clinics (six total pediatric rheumatologists) in Ohio and Kentucky, with each site having Institutional Review Board approval. All participants met Yunus and Masi criteria [3] adapted for JFM classification which includes: generalized musculoskeletal aching for greater than three months, the presence of at least 5 out of 18 tender points, and at least three associated symptoms such as poor sleep quality, fatigue, chronic anxiety, irritable bowel syndrome or chronic headaches. Participants had to have an average pain intensity of at least 4 (on a 0-10 cm visual analog scale, VAS) to be eligible for the trial and were excluded if they had other chronic rheumatic diseases such as juvenile idiopathic arthritis or systemic lupus erythematosus or other comorbid illness that could cause fibromyalgia-like symptoms (e.g. thyroid disease).

### Procedure

Participants were informed of the study by their primary rheumatologist and contacted by a research assistant for their interest in participation. Written informed consent from parents and assent from adolescents was obtained. Participants were asked to complete a daily pain diary for the week prior to the initial evaluation. All participants were formally evaluated by a pediatric rheumatologist with a complete medical history and physical examination.

### Measures

#### Demographic information

A demographic form regarding the participant’s age, sex, race and ethnicity was completed by the parent(s).

#### Tender point assessment (pain sensitivity)

An 18-count TP examination, as described in the ACR criteria for FM [4], was performed by a trained pediatric rheumatologist. A dolorimeter (Pain Diagnostics & Treatment Inc., Great Neck, NY) with a 1 cm rubber tip was applied at a rate of 1 kg/cm^2^ of pressure per second. The participant was asked to inform the evaluator the point at which pain (not pressure) was felt and this pain threshold, from 1 to > 4 kg/cm^2^, was noted for each of the 18 TP sites. An average TP score (pain threshold) based upon the 18 points was calculated, with lower scores indicating greater pain sensitivity. The total number of positive (score of < 4 kg/cm^2^) painful TPs was also recorded.

#### Pain rating (VAS)

For one week prior to their assessment visit, adolescents completed a diary rating of their average level of pain each day using a Visual Analog Scale (VAS, 10 cm horizontal line with no numerical markings). VAS scales [16] are well-validated and widely used in pediatric pain research [17]. The pain VAS scale was anchored with the descriptors of 0 = “no pain” and 10 = “worst possible pain”. The average pain rating over the period of one week of daily diaries was used as a measure of self-reported clinical pain intensity.

#### Medical chart review

Medical charts were reviewed for participant information regarding physical exam findings, including joint HM. HM was defined by each individual clinician’s assessment of increased joint laxity (HM noted to be present if found in at least 4 joints). For this preliminary clinical observational study, information about specific standardized criteria for HM (such as Beighton scores) was not available due to variability in clinical documentation in medical records at each site. However, all examining physicians were board certified/eligible pediatric rheumatologists who are trained to use similar criteria for classification of HM in children. Each of the 6 pediatric rheumatologists indicated they used either the Beighton [18] or Carter and Wilkinson [19] criteria (Table 1) for joint hypermobility and documented hypermobility if they met criteria.

**Table 1:** Criteria for Joint Hypermobility

Criteria	Definition	Scoring
Beighton [18]	Passive hyperextension ≥10 degrees of the knee	
		Right – 1 point
		Left –1 point
	Passive hyperextension ≥10 degrees of the elbow	Right – 1 point
		Left – 1 point
	Passive apposition of the thumb to the flexor aspect of the forearm	Right – 1 point
		Left – 1 point
	Passive dorsiflexion of 5^th^ finger metacarpophalangeal joint to ≥ 90^o^	Right – 1 point
		Left – 1 point
	Forward flexion of the trunk, with the knees straight, so that the palms rest easily and flat on the floor	1 point
	**A score of 4/9 or greater equates hypermobility	
Carter & Wilkinson [19]	Bilateral passive apposition of the thumb to the flexor aspect of the forearm	1 point
	Bilateral passive hyperextension of the fingers to lie parallel with the forearm	1 point
	Passive hyperextension of the elbows > 10^o^	1 point
	Passive hyperextension of the knees > 10^o^	1 point
	Bilateral excessive passive dorsiflexion of ankle and excessive foot eversion	1 point
	**A score of 3/5 or greater equates hypermobility	

### Statistical analyses

All data were entered and analyzed using SPSS Version 15.0 software. Descriptive data on pain VAS scores, average TP sensitivity (TP threshold score) and number of painful tender points (TP count) were computed. Pearson correlation coefficients were computed to assess the relationship between average VAS pain score, average TP score, and TP count. Average pain VAS and TP scores were compared between the HM+ and HM- groups using t-tests, and TP counts in the HM+ versus HM- groups were compared using the non-parametric Mann-Whitney test (due to non-normal distributed data on TP counts).

## Results

### Self-reported pain and pain sensitivity

**Figure 1:**
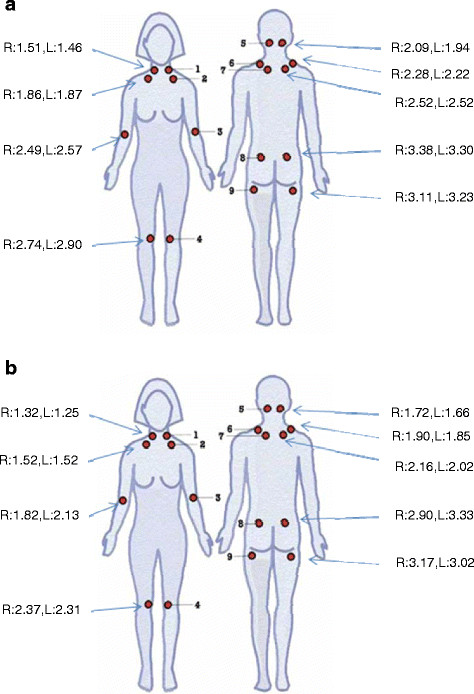
**a. Tender point averages in JFM patients without joint HM. b**. Tender point averages in JFM patients with joint HM. Legend Figure 1 a/b. *Distribution of mean threshold scores (kg/cm*^*2*^*) by tender point location.* Mean tender point scores are noted for each of the 18 tender point sites. R: Right, L: Left, 1. Low Cervical, 2. Anterior Rib, 3. Lateral Epicondyle, 4. Medial Fat Pad, 5. Occiput, 6. Trapezius, 7. Supraspinatus, 8. External Outer Gluteal, 9. Greater Trochanter.

Among the 131 participants, the mean pain rating as measured by daily diaries on a 10-point VAS was 5.73 (SD 1.37). The mean TP count among all participants was 16.23 (SD 2.30) with 85% having 14 or more positive TPs. The mean TP score was 2.26 kg/cm^2^ (SD 0.58) (Table 2). Among the 18 TP sites, TP locations with the lowest mean pain threshold were in the head and neck region (anterior rib, low cervical, and occiput) with lower average thresholds consistently seen among the HM group (Figure 1A/B). In correlations between self-reported VAS and pain sensitivity measures based upon dolorimetry, neither TP score (Pearson r = -0.08) nor TP count (Spearman ρ = 0.06) was significantly associated with VAS self-report of pain intensity (Table 3).

**Table 2:** Demographic information and mean pain scores (self-report VAS, tender point total and scores)

Characteristic	Mean	SD^a^	Range
Age (years)	15.08	1.81	11-18
VAS^b^ Rating (0-10)	5.73	1.37	1.16-8.86
Number of Positive Tender Points^c^ (0-18)	16.23	2.30	9-18
Tender Point Score (1-4 kg/cm^2^)	2.26	0.58	
	**Number**	**%**	
Female	121	92.4	
Male	10	7.6	
Race			
Caucasian	117	89.3	
Black or African-American	9	6.9	
Other	5	3.8	

^a^ SD – standard deviation, ^b^ VAS – Visual Analogue Scale, ^c^ A positive tender point equates a dolorimetry score of < 4 kg/cm^2^ of pressure.

**Table 3:** Correlation analysis of the relationship between mean VAS pain score, tender point score, and tender point count

	Tender Point Score	Tender Point Count	VAS Rating
Tender Point Score	1	−0.84^b^	−0.08
Tender Point Count	−0.84^b^	1	0.06
VAS^a^ Rating	−0.08	0.06	1

^a^ VAS – Visual Analogue Scale, ^b^ p-value <0.001.

### Role of hypermobility in the pain experience

Documentation of HM was available for 95% (122/131) of participants. Of the 122 JFM patients, nearly half (48%, n = 58) were noted by their primary rheumatologist to have hypermobile joints. Mean self-reported VAS in the HM+ group was 5.59 (SD 1.41) compared to 5.79 (SD 1.28) in the HM- group (p = 0.42, not significant). However, the HM+ patients had significantly greater pain sensitivity with lower mean TP scores (2.10 kg/cm^2^ vs. 2.41 kg/cm^2^, p = 0.002) and higher TP count (16.77 vs. 15.72, p = 0.003) than HM- patients (Table 4).

**Table 4:** Relationship of mean VAS score, tender point count and tender point score among JFM patients with or without joint hypermobility

	Hypermobility	No Hypermobility		
	Mean	Mean	P^b^	CI^c^
VAS^a^ Rating	5.59	5.79	0.42	−2.92 – 0.68
Tender Point Count	16.77	15.72	0.003	−1.9 – -0.24
Tender Point Score	2.10	2.41	0.002	0.12 – 0.52

^a^ VAS – Visual Analogue Scale, ^b^ – Significant p-value <0.05, ^c^ – Confidence Interval.

## Discussion

Fibromyalgia syndrome in adolescents is characterized by chronic widespread musculoskeletal pain and multiple associated symptoms. Consistent with prior reports [8,9], results of this study showed that joint HM commonly co-occurs with JFM in children and adolescents with nearly half of the adolescents with JFM also having HM. This is similar to findings from adult fibromyalgia studies which have reported that 46.6% [20] to 62% [10] of fibromyalgia patients also had HM. In addition to replicating findings regarding the overlap between JFM and HM, results of this study suggest the possibility that a ‘benign’ condition like joint laxity can be associated with enhanced pain sensitivity in JFM patients. Specifically, HM+ patients show significantly greater physiologic sensitivity as measured by TP threshold and TP count than HM- patients, even though their self-report of clinical pain intensity did not differ. However, it is unclear if this difference is clinically relevant as all patients had relatively high pain sensitivity. Interestingly, all tender point locations were lower among the HM+ group and not significantly different among areas that are typically flexible (i.e. knees).

Potential mechanisms underlying the relationship between HM and pain sensitivity in JFM patients might include genetic vulnerability associated with gene polymorphisms responsible for pain perception [21][22], immunologic factors [23], or related to the common features of dysautonomia (syncope, orthostatic hypotension, tachycardia, etc.) often reported by both HM and JFM patients [24,25]. Interestingly, despite the evidence of increased pain sensitivity in JFM HM+ adolescents, we found that the subjective report of clinical pain (VAS pain ratings) did not correlate with physiologic pain sensitivity. Furthermore, there was no significant difference in clinical pain reports between JFM adolescents with and without HM. These results reinforce the notion that pain is a complex subjective multidimensional experience. Results obtained from different assessment methods (subjective pain ratings versus sensory testing) may therefore represent different facets of pain.

The results of this study have implications for the measurement of pain outcomes in clinical trials of JFM. In recent studies, cognitive behavioral therapy (CBT) has been found to be a promising treatment for JFM [26,27] and the parent clinical trial associated with this study showed that CBT was effective in reducing pain-related disability and depressive symptoms. Patients also reported reduced pain intensity (VAS) levels, but there was no change in tender point sensitivity after CBT. In order to change physiologic pain sensitivity, other types of interventions, for example, intensive aerobic exercise programs, which have been shown to be effective for pain reduction [28] need to be further studied. Tailored programs for JFM HM+ children focusing on joint protection and strengthening might also be investigated to see if they can produce reductions in mechanical stress which could ameliorate heightened pain sensitivity.

We recognize several limitations of our study. Our patients were recruited from tertiary pediatric rheumatology clinics; therefore, they may represent the most severe and prolonged cases of JFM. The majority of patients (85%, n = 112) had a total of >14 positive TP and nearly half (47%) had 18/18 positive TP. Another limitation is that the scoring systems for HM (i.e. Beighton, Brighton, Carter and Wilkinson) were not strictly standardized in our study; however, each rheumatologist indicated they documented hypermobility if a patient met criteria. Despite the potential recruitment of patients with more severe JFM and the non-standardized clinician assessment of HM, the prevalence of HM in this sample was found to be similar to that reported by Siegel and colleagues, i.e., almost half of the JFM sample [9].

## Conclusions

The findings of this study strengthen prior reports of joint HM being commonly observed among clinical populations of adolescent JFM patients. Additionally, we found that HM is associated with heightened pain sensitivity. Suggestions for future research include identifying the genetic link(s) attributable to these associated conditions, continued physiological assessments to better understand the mechanisms of pain in both HM and JFM, and evaluation of targeted exercise programs for this population. A greater understanding of the various aspects of pain in JFM is needed to further enrich the multidisciplinary approach for treatment of this complex syndrome.

## Competing interests

The authors declare that they have no competing interests.

## Authors’ contributions

TT conceived of the study, and participated in its design and coordination, acquisition of data, analysis and data interpretation and helped to draft the manuscript. AD participated in data entry as well as performed the statistical analysis. KS participated in acquisition of data and interpretation. SS participated in acquisition of data and interpretation. PH participated in acquisition of data and interpretation. SKZ conceived of the study, and participated in its design and coordination and helped to draft the manuscript. All authors read and approved the final manuscript.

### Funding

This work was supported by a grant from the National Institute of Arthritis and Musculoskeletal and Skin Diseases [Grant # R01 AR050028].
